# Trans-allelic mutational effects at the *Peg3* imprinted locus

**DOI:** 10.1371/journal.pone.0206112

**Published:** 2018-10-18

**Authors:** Corey L. Bretz, Joomyeong Kim

**Affiliations:** Department of Biological Sciences, Louisiana State University, Baton Rouge, LA, United States of America; Harvard Medical School, UNITED STATES

## Abstract

How one allele interacts with the other for the function of a gene is not well understood. In this study, we tested potential allelic interaction at the *Peg3* imprinted locus with several mutant alleles targeting an Imprinting Control Region, the Peg3-DMR. According to the results, maternal deletion of the Peg3-DMR resulted in 2-fold up-regulation of two paternally expressed genes, *Peg3* and *Usp29*. These trans-allelic mutational effects were observed consistently throughout various tissues with different developmental stages. These effects were also associated mainly with the genetic manipulation of the Peg3-DMR, but not with the other genomic changes within the *Peg3* locus. The observed trans-allelic effects were unidirectional with the maternal influencing the paternal allele, but not with the opposite direction. Overall, the observed mutational effects suggest the presence of previously unrecognized trans-allelic regulation associated with the Peg3-DMR.

## Introduction

In diploid organisms, the genotypes of two alleles dictate the phenotypes for a given locus. In many cases of Mendelian genetics, two alleles tend to be additive and independent in terms of functional contribution to the total amount of mRNA and protein products. In some cases, however, two alleles are not simply additive, but rather functionally influence each other, either positively or negatively, resulting in much less or more functional output from one allele relative to the averaged output of two alleles [1]. This functional interaction or dependency between two alleles has been often observed in traditional quantitative genetics and further substantiated through modern genetic tools, such as molecular cytogenetics and genomic approaches [2–4]. This allelic interaction is known to be prevalent among many different types of genes. In the case of imprinted genes, introduction of mutations into one allele often results in unexpected outcomes in the expression and DNA methylation levels of the second allele. This type of trans-allelic mutational effect has been previously reported from the *Snrpn*/*Ube3a*, *H19*/*Igf2* and *Rasgrf1* imprinted domains [5–8]. The observed trans-allelic effects or interactions are thought to be mediated through several modes, including transvection and paramutation-like modes [9–11]. However, the detailed mechanisms are currently unknown.

*Peg3* (paternally expressed gene 3) is the founding member of the 500-kb imprinted domain localized in the proximal mouse chromosome 7/ human chromosome 19q13.4 [12–14]. This domain contains paternally expressed *Peg3*, *Usp29*, *Zfp264*, *APeg3*, and maternally expressed *Zim1*, *Zim2*, *Zim3* [15]. The imprinting and transcription of this domain is regulated through the Peg3-DMR (Differentially Methylated Region), a 4-kb genomic interval encompassing the bidirectional promoter for *Peg3* and *Usp29* [16, 17]. This DMR obtains oocyte-specific DNA methylation through currently uncharacterized mechanisms involving an upstream alternative promoter, U1 [18]. Thus, deletion of the U1 promoter usually results in complete loss of DNA methylation on the Peg3-DMR, causing loss of imprinting throughout the entire *Peg3* domain [18]. Paternal deletion of the Peg3-DMR itself also causes similar domain-wide effects as an ICR, including complete abrogation of the transcription of two paternally expressed genes, *Peg3* and *Usp29*, and subsequent bi-allelic expression of the adjacent imprinted genes, *Zim1* and *Zim2* [16, 17]. In contrast, maternal deletion of the Peg3-DMR was not initially thought to have any effect, since its maternal allele is already repressed by DNA methylation and thus presumably non-functional. Yet, maternal deletion of this ICR caused an unexpected outcome, up-regulating the expression levels of two paternally expressed genes, *Peg3* and *Usp29*, from the opposing paternal allele [17]. This unexpected trans-allelic outcome was also accompanied with a boost in growth and survival rates among the animals, consistent with the up-regulation of *Peg3* and *Usp29*.

In the current study, we further characterized the trans-allelic effects observed from the *Peg3* locus with several sets of breeding experiments involving various mutant alleles. According to the results, the observed effects appeared to be consistent throughout various tissues and also during the different stages of development. The trans-allelic effects were also associated mainly with the genetic changes to the genomic interval spanning the Peg3-DMR.

## Results

### Various mutant alleles targeting the imprinted *Peg3* locus

**Fig 1:**
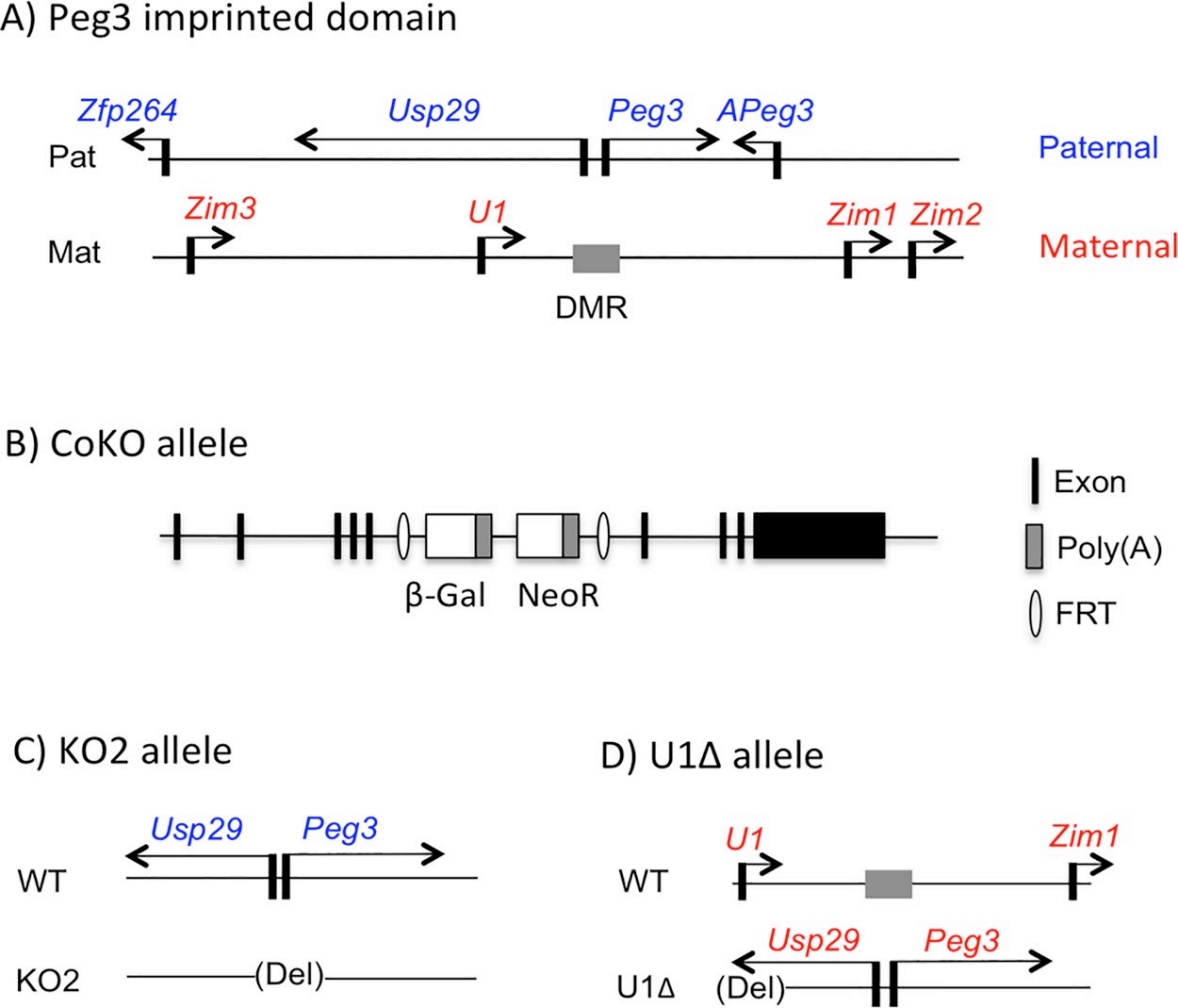
Various mutant alleles targeting the *Peg3* imprinted locus. (**A**) Genomic structure of the mouse *Peg3* imprinted domain. The paternally and maternally expressed genes are indicated with blue and red, respectively. The transcriptional direction is indicated with an arrow, while the methylated maternal allele of the Peg3-DMR is indicated with a grey box. (**B**) Schematic representation of the CoKO allele. The exons of *Peg3* are indicated with filled boxes, while the two reporters within the 7-kb inserted expression cassette are indicated with open boxes. (**C**, **D**) Schematic representation of KO2 and U1Δ alleles. The deleted region for each mutant allele is indicated with a parenthesis.

In the past, several mutant alleles targeting the *Peg3* locus have been generated to characterize the regulatory mechanisms governing the imprinting and function of this mouse locus (**Fig 1**). First, the mouse *Peg3* locus was mutated through inserting a 7-kb exogenous cassette expressing β-galactosidase (*β-Gal*) and neomycin resistance gene (*NeoR*) into the 5th intron [19, 20]. Paternal transmission of this allele, named CoKO (Conditional KnockOut-ready), expresses the β-Gal protein under the control of the endogenous promoter of *Peg3*, which has been used for monitoring the expression patterns of *Peg3* (**Fig 1B**). In this mutant allele, the transcription of *Peg3* becomes truncated due to two Poly-A signals included in the expression cassette, thus allowing us to simultaneously characterize the mutational effects of *Peg3* [19, 20]. For the current study, the CoKO allele has been used as a proxy allele reporting the expression profile of *Peg3*. Second, the 4-kb genomic interval covering the Peg3-DMR has been deleted to test its predicted function as an ICR, named KO2 (**Fig 1C**). Paternal transmission of this mutant allele has been previously characterized, showing complete abolition of the transcription of *Peg3* and *Usp29* [17]. Maternal transmission of this mutant allele was further characterized in the current study. Third, the 1-kb genomic interval covering the alternative promoter U1 has been also deleted, named U1Δ (**Fig 1D**). Maternal transmission of this allele usually results in complete loss of DNA methylation at the Peg3-DMR allowing expression of *Peg3* and *Usp29* from the normally silenced maternal allele [18]. The maternal transmission of this allele was used to activate the maternal allele of *Peg3* and *Usp29* in the current study.

### Deletion effects of the maternal allele on the paternal allele of *Peg3*

**Fig 2:**
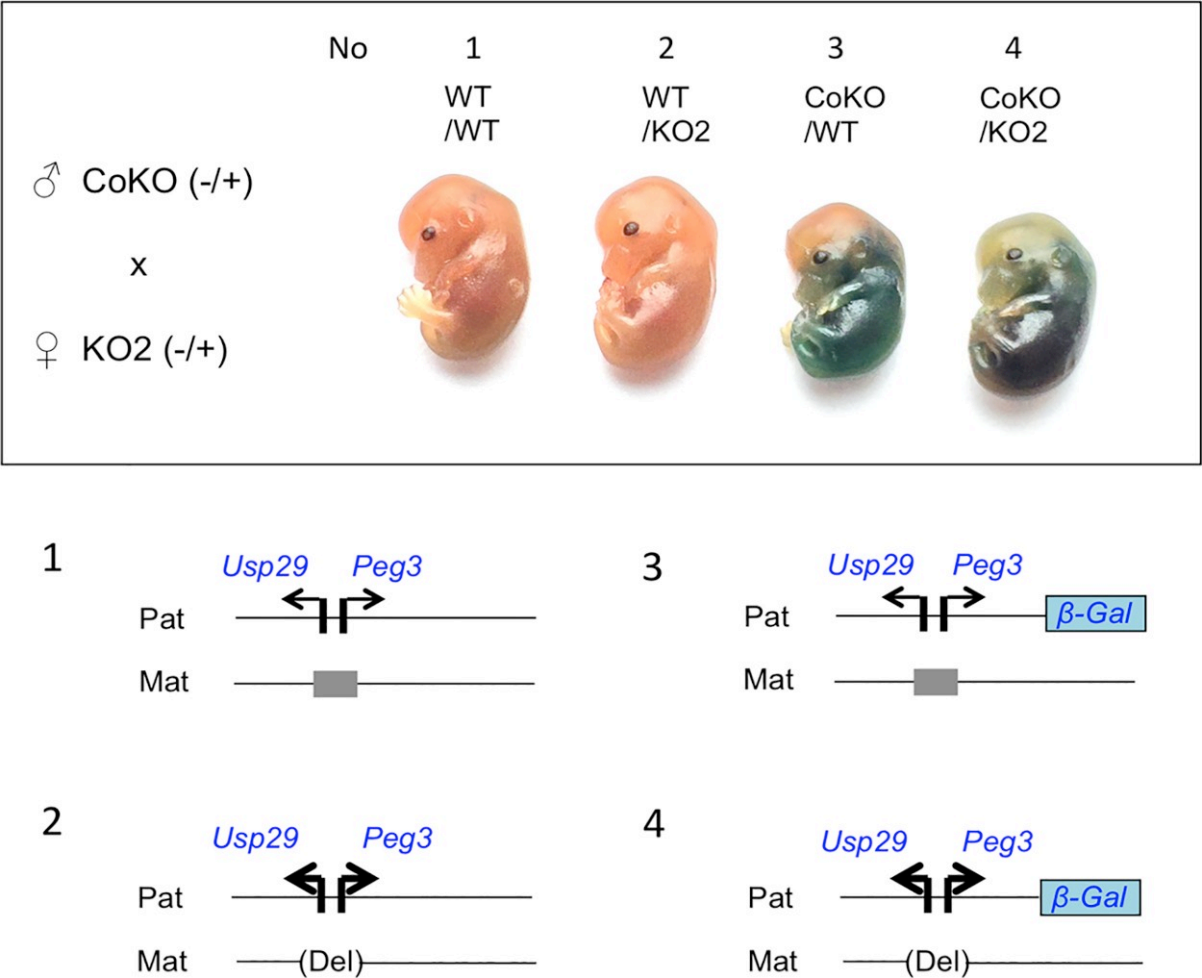
Deletion effects of the maternal allele on the paternal allele of *Peg3*. The 14.5-dpc embryos derived from the crossing between male heterozygotes for *Peg3*^*CoKO/+*^ and female heterozygotes for *Peg3*^*KO2/+*^ were analyzed with whole-mount β-Gal staining. The upper panel shows the representative images derived from a set of embryos with four possible genotypes, No1 through No4. The bottom panel illustrates the schematic representation of the two alleles for each genotype.

The following set of breeding experiments was performed to test potential effects of the maternal deletion on the paternal allele of the Peg3-DMR. Four male heterozygotes for *Peg3*^*CoKO/+*^ were mated with 8 female heterozygotes for *Peg3*^*KO2/+*^ (**Fig 2**). This set of breeding experiments generated 9 litters of 72 pups, and the average litter size, 8 pups per litter, appeared to be normal given the C57BL/6J genetic background. Also, the ratio among the four genotypes was close to the expected mendelian ratio: WT/WT: WT/KO2: CoKO/WT: CoKO/KO2 = 17: 21: 20: 14. Thus, no embryonic lethality was likely associated with this breeding scheme. Four females were further used for timed mating to harvest 14.5-dpc (days postcoitum) embryos, providing three sets of the embryos with four possible genotypes as indicated with No1-4 in **Fig 2**. Each set of embryos were used for whole-mount β-Gal staining. As shown in **Fig 2**, two genotypes, No1 and No2, did not carry the CoKO allele, thus no visible staining by the β-Gal activity. However, we did observe size variation between these two types: No2 embryos were usually bigger than No1 embryos. This may be an indication that the expression levels of *Peg3* are likely greater in No2 than in No1 due to the maternal deletion of the Peg3-DMR. This is consistent with the previous observation that the maternal deletion of the Peg3-DMR resulted in increased growth and survival rates among the animals [17]. On the other hand, the other two genotypes, No3 and No4, displayed readily detectable levels of the β-Gal activity. Detailed inspection also indicated different levels of the β-Gal activity: the levels observed from No4 were much greater than those from No3. The greater levels were also observed uniformly throughout the entire body of the tested embryos. Again, this may be an indication that the promoter activity of the paternal allele of *Peg3* in No 4 is greater than in No3 due to the maternal deletion of the Peg3-DMR. We repeated this series of staining experiments three times with the three sets of harvested embryos, which provided a consistent outcome: the two genotypes, No2 and No4, with the maternal deletion of the Peg3-DMR exhibiting increased activity of the paternal allele.

### Activation effects of the maternal allele on the paternal allele of *Peg3*

**Fig 3:**
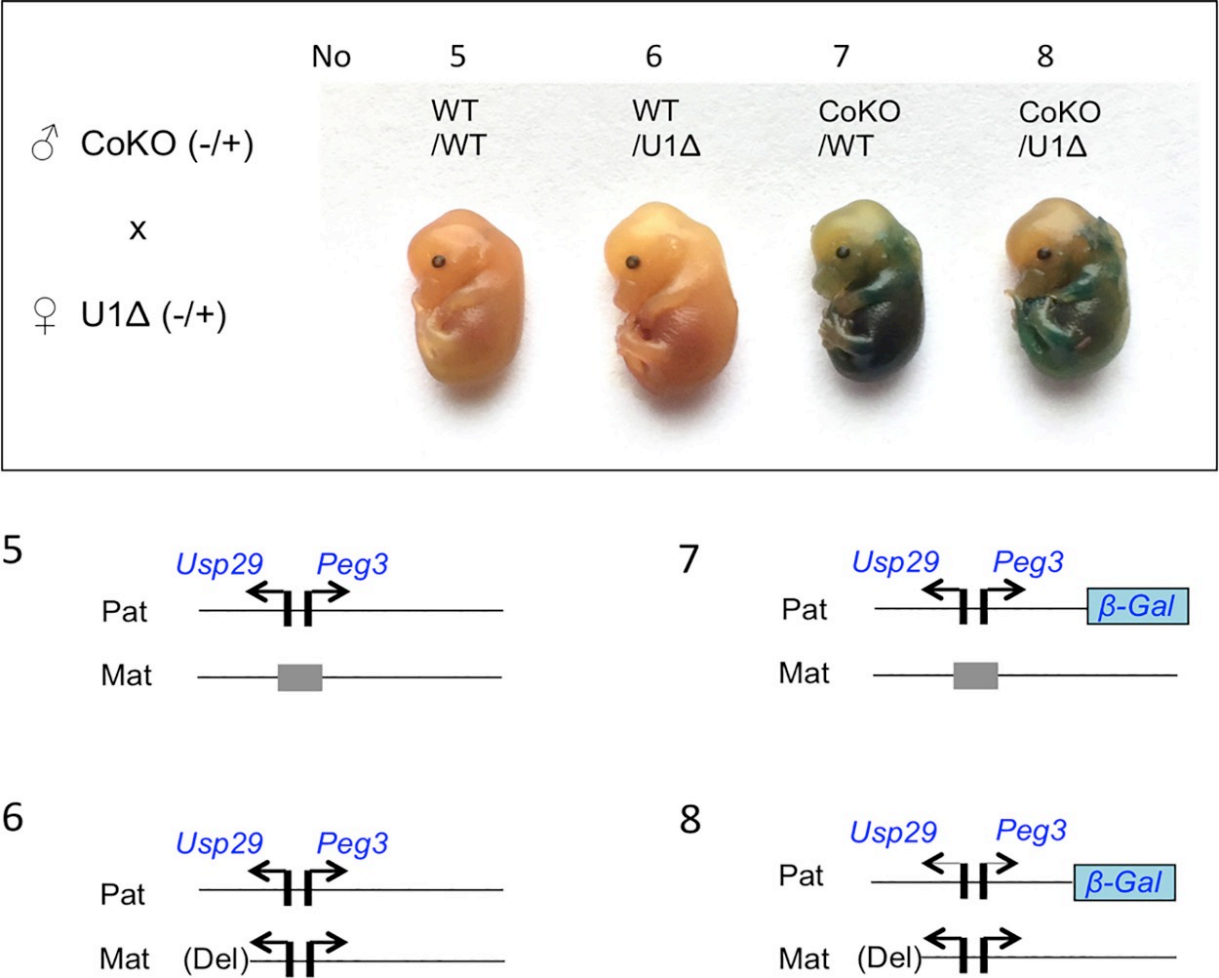
Activation effects of the maternal allele on the paternal allele of *Peg3*. The 14.5-dpc embryos derived from the crossing between male heterozygotes for *Peg3*^*CoKO/+*^ and female heterozygotes for *Peg3*^*U1Δ/+*^ were analyzed with whole-mount β-Gal staining. The upper panel shows the representative images derived from a set of embryos with four possible genotypes, No5 through No8. The bottom panel illustrates the schematic representation of the two alleles for each genotype.

We also performed the following set of breeding experiment to test potential effects of activating the maternal allele on the activity of the paternal allele. Two male heterozygotes for *Peg3*^*CoKO/+*^ were mated with 4 female heterozygotes for *Peg3*^*U1Δ/+*^ (**Fig 3**). This set of breeding experiments generated 5 litters of 39 pups, and the average litter size, 7.8 pups per litter, also appeared to be normal given the C57BL/6J genetic background. The ratio among the four genotypes was close to the mendelian ratio: WT/WT: WT/U1Δ: CoKO/WT: CoKO/U1Δ = 11: 12: 7: 9. Thus, this also indicated no major embryonic lethality associated with this breeding scheme. Two females were used for timed mating to derive 14.5-dpc embryos, providing two sets of the embryos with four possible genotypes, as indicated as No5-8 in **Fig 3**. These two sets of embryos were also used for whole-mount β-Gal staining. Similar to the patterns described above, the two genotypes, No5 and No6 without the CoKO allele, were not stained at all, but showed size variation between the two genotypes: No6 were larger than No5 embryos. This may have been contributed by No6 embryos having double dosage of *Peg3* and *Usp29* due to the deletion of the U1 promoter activating the maternal allele [18]. On the other hand, the two remaining genotypes, No7 and No8, showed high levels of the β-Gal activity. Yet, the activity observed from No8 was slightly lower than that from No7, indicating that the promoter activity of the paternal allele in No8 may have been down-regulated as compared to that of No7. These patterns were reproducible between the two sets of the harvested embryos. Thus, activation of the maternal allele appeared to slightly down-regulate the promoter activity of the paternal allele of the Peg3-DMR.

### Effects of various mutant maternal alleles on the paternal allele of *Peg3*

**Fig 4:**
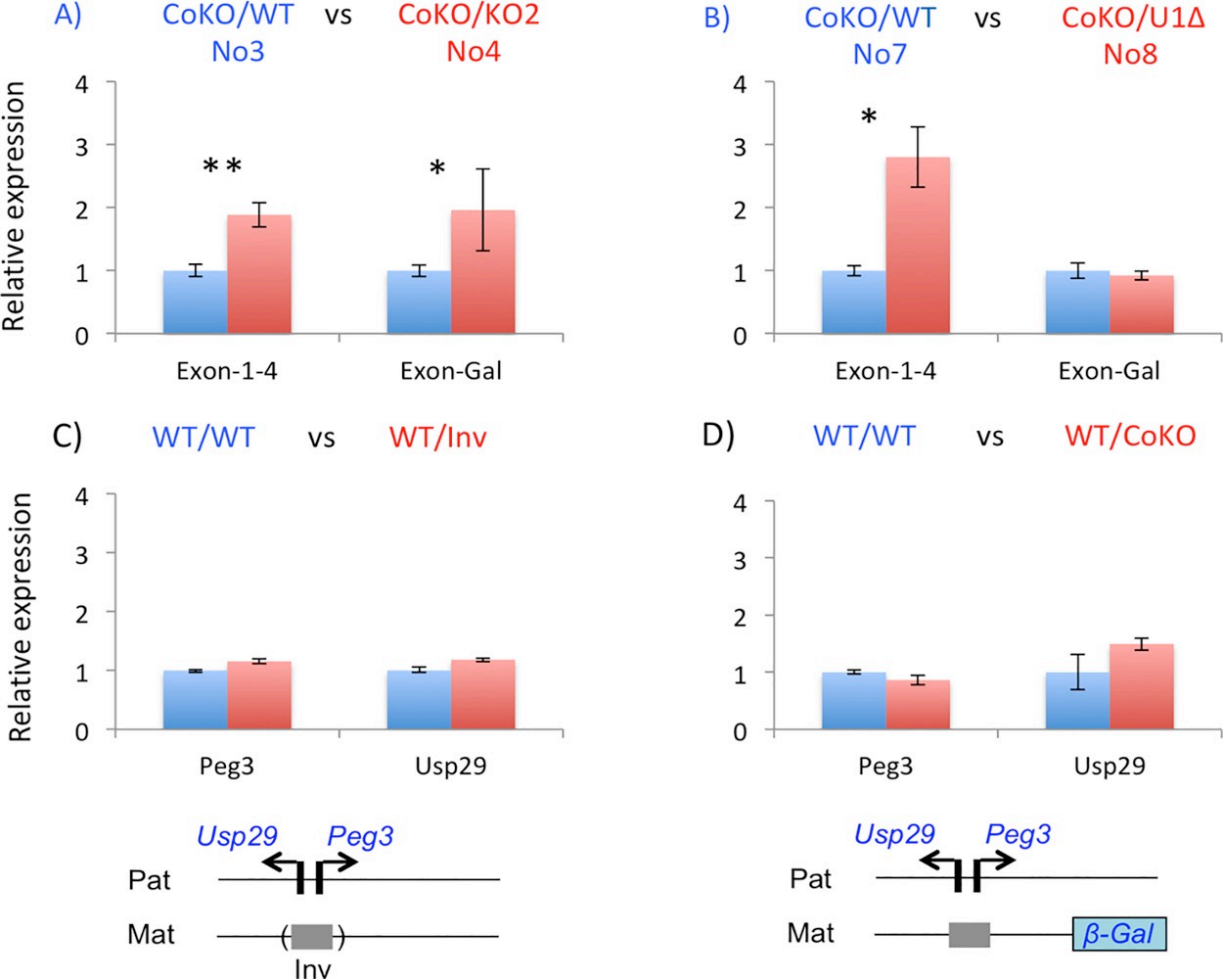
Effects of the mutant maternal alleles on the paternal allele of *Peg3*. Total RNA isolated from the one-day-old neonates of four breeding schemes was used to measure mutational effects on the expression levels of the imprinted genes. For this comparison, expression levels of each gene were first normalized with those of β-actin, which were then further compared between two neonates: the pups with blue serve as a control, while the pups with red are the testing subjects with the maternal transmission of a mutant allele. The genotype for each sample is indicated on top in the following manner, paternal/maternal. Schematic representation for the two alleles of each sample is shown below (**C**, **D**). The schematic representation for two sets (**A**, **B**) are shown in **Fig 2** and **Fig 3**, respectively. Statistical significances for each comparison are indicated with * (p < 0.05) or ** (p < 0.01).

The results described above were further tested through a series of independent expression analyses (**Fig 4**). Total RNA was isolated from one-day-old pups, which were then used for cDNA synthesis. These cDNA were subsequently used for qRT-PCR analyses. First, potential up-regulation of *Peg3* was tested through comparing the expression levels of the fusion transcript from the CoKO allele between No3 (CoKO/WT) and No4 (CoKO/KO2) samples (**Fig 4A**). Two separate regions were selected to measure the expression levels: the first amplicon covering Exon1-4 of *Peg3* and the second amplicon covering the coding region of β-Gal, Exon-Gal (**Fig 1B**). The results from these two amplicons indicated that the expression levels of the CoKO allele in No4 were two-fold higher than those from No3, which agrees with the observation derived from whole-mount β-Gal staining (**Fig 2**). Second, we also performed a similar series of qRT-PCR analyses with a set of the total RNA isolated from the following one-day-old neonates: No7 (CoKO/WT) and No8 (CoKO/U1Δ) (**Fig 4B**). The results derived from the amplicon Exon1-4 indicated that the expression levels of *Peg3* were 2.8-fold higher in No8 than in No7. Since this amplicon was designed to measure the expression from both the endogenous and the CoKO alleles, the increased levels detected in No8 most likely represent the added contribution from the de-repressed maternal allele of *Peg3* by the deletion of U1. On the other hand, the amplicon unique to the CoKO allele, Exon-Gal, showed slightly lower levels in No8 (92%) than in No7. Although statistically insignificant (*p* = 0.1542), the slightly lower levels observed from No8 was again consistent with the results from whole-mount β-Gal staining (**Fig 3**). This series of expression analyses were repeated three times with biological replicates, and the results were reproducible. Overall, the up-regulation observed from No4 and the slight down-regulation observed from No8 were consistent with the results from whole-mount β-Gal staining experiments. It is salient to note that similar conclusions have been derived from two different-stage samples, embryonic and neonatal tissues, with two independent approaches.

We further tested potential trans-allelic effects using another mutant allele targeting the Peg3-DMR (**Fig 4C**). We have recently generated this mutant allele, in which the orientation of the 4-kb genomic interval of the Peg3-DMR has been inverted relative to that of the surrounding regions [21]. The paternal transmission of this allele resulted in 9-fold down-regulation of *Peg3*, but 2-fold up-regulation of *Usp29*. In contrast, the maternal transmission did not cause any change in DNA methylation on the Peg3-DMR. We performed a similar series of expression analyses using a set of total RNA isolated from the following two types of neonates: WT/WT and WT/Inv. The results indicated no major difference between these two samples, suggesting that the inversion of the Peg3-DMR may not have interfered the trans-allelic effects at this locus. This further suggests that the orientation of the Peg3-DMR may not be a critical factor to the observed trans-allelic interaction. Finally, we also tested whether the observed trans-allelic effects are detectable with genomic changes other than the deletion of the Peg3-DMR (**Fig 4D**). In this study, the CoKO allele has been used as a proxy for the paternal allele, but this allele in fact contains a 7-kb exogenous sequence 13-kb downstream of the Peg3-DMR, which might cause some impact on the unknown functions of the maternal allele. Thus, we used the neonates with the maternal transmission of the CoKO allele (WT/WT versus WT/CoKO) to gauge the possibility of confounding effects. According to the results, the insertion of this 7-kb exogenous construct did not cause any effects on the expression levels of *Peg3* and *Usp29* from the paternal allele. Thus, this suggests that the observed trans-allelic effects at the *Peg3* locus may be unique to some changes that are associated with the genomic interval covering the Peg3-DMR.

### Deletion effects of the paternal allele on the maternal allele of *Peg3*

**Fig 5:**
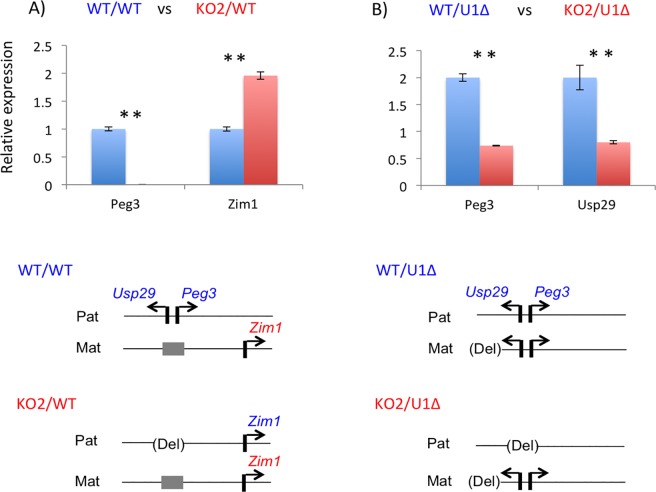
Deletion effects of the paternal allele on the maternal allele of *Peg3*. Total RNA isolated from the one-day-old neonates of two breeding schemes was used to measure mutational effects on the expression levels of the imprinted genes (**A**, **B**). For this comparison, expression levels of each gene were first normalized with those of β-actin, which were then further compared between two neonates: the pups with blue serve as a control, while the ones with red are the testing subjects with the paternal transmission of a mutant allele. The genotype for each sample is indicated on top in the following manner, paternal/maternal. Schematic representation for the two alleles of each sample is shown below. Statistical significances for each comparison are indicated with * (p < 0.05) or ** (p < 0.01).

We also tested potential effects of the paternal Peg3-DMR deletion on the maternal allele activity (**Fig 5**). As described above, several series of qRT-PCR analyses were conducted also with the total RNA isolated from the following sets of neonates. First, we compared the expression levels of the genes between the two genotypes: WT/WT and KO2/WT (**Fig 5A**). Since the maternal alleles of *Peg3* and *Usp29* are already silenced by DNA methylation, measuring their expression levels was not a feasible option to detect potential deletion effects of the paternal allele of the Peg3-DMR. As an alternative, we measured the expression levels of an adjacent gene, *Zim1*. The expression levels of *Zim1* were 2.0-fold up-regulated in KO2/WT relative to those from WT/WT. We have previously reported that paternal deletion of the Peg3-DMR usually causes paternal activation and subsequent bi-allelic expression of *Zim1* [17, 22]. Thus, the observed 2.0-fold up-regulation may reflect the combined contribution from both alleles with each being equally 1.0-fold [17, 22]. This further indicated that the expression levels of the maternal allele of *Zim*1 were not affected in response to the paternal deletion of the Peg3 -DMR. Therefore, we concluded that there were no obvious trans-allelic effects on *Zim1* by the paternal deletion of the Peg3-DMR. Second, we also repeated a similar series of expression analyses using the total RNA from the following two genotypes: WT/U1Δ and KO2/U1Δ (**Fig 5B**). In this set of neonates, the maternal allele of *Peg3* and *Usp29* are activated by the deletion of U1, thus allowing us to directly measure potential effects of deleting the paternal allele on the maternal allele activity of the Peg3-DMR. According to the results, the relative expression levels of *Peg3* and *Usp2*9 were 38 and 40%, respectively, in KO2/U1Δ relative to those from WT/U1Δ. These levels of the down-regulations appeared to be slightly steeper than expected, since removing one active allele should have resulted in a 50% reduction, if there was no positive or negative interaction between two alleles. The difference, however, appeared to be too marginal to be further investigated, since this level of variation could stem from the experimental setup. Overall, these two series of expression analyses concluded that paternal deletion of the Peg3-DMR did not cause any major trans-allelic effects on the maternal allele activity of the Peg3-DMR. We repeated this series of analyses with two biological replicates, which provided similar outcomes as presented.

## Discussion

In the current study, we tested potential trans-allelic effects at the *Peg3* imprinted locus by analyzing the expression levels of the imprinted genes in several mutant models. According to the results, maternal deletion of the Peg3-DMR caused the most significant effects on the activity of the paternal allele, 2-fold up-regulation of *Peg3* and *Usp29*. The observed trans-allelic effects were also detected consistently throughout the various tissues and also during the different stages of development. These effects were detectable mainly with the genomic deletion of the Peg3-DMR, but not with the other genomic changes so far. Also, the observed trans-allelic effects were unidirectional with the maternal allele influencing the gene activity of the paternal allele, but not with the other direction. Overall, this suggests the presence of an unknown trans-allelic regulatory mechanism associated with the Peg3-DMR.

**Fig 6:**
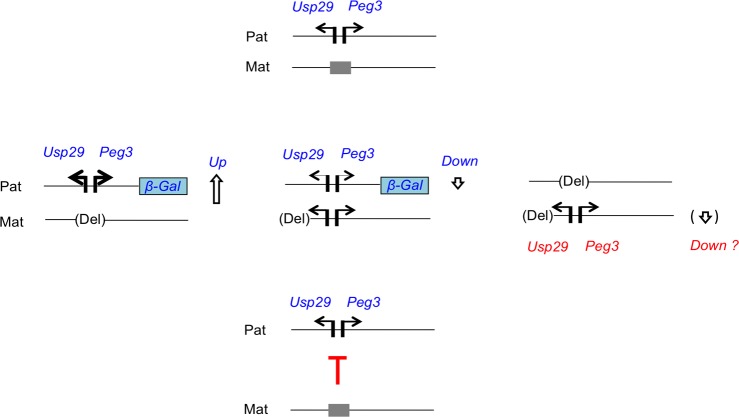
Trans-allelic mutational effects observed at the *Peg3* imprinted locus. The schematic representation on top illustrates the paternal and maternal alleles of the *Peg3* locus: the two paternally expressed genes, *Peg3* and *Usp29*, are indicated with two arrows, whereas the methylated maternal allele of the Peg3-DMR is indicated with a grey box. The three representations on middle summarize the observed trans-allelic mutational effects: 1) deletion of the maternal allele of the Peg3-DMR causing up-regulation of the paternal allele, 2) activation of the maternal allele of the Peg3-DMR by the deletion of U1 causing slight down-regulation of the paternal allele, and 3) deletions of the paternal allele of the Peg3-DMR causing either no or slight down-regulation of the maternal allele. The first one appears the most consistent and robust among these potential trans-allelic effects. Given this conclusion, the bottom panel hypothesizes potential suppressor roles played by the maternal allele for the regulation of the paternal allele.

The trans-allelic mutational effects observed at the *Peg3* locus can be summarized and further interpreted in the following manner (**Fig 6**). First, maternal deletion of the Peg3-DMR resulted in 2-fold up-regulation of the two paternally expressed genes, *Peg3* and *Usp29* (**Fig 2** and **Fig 4A**). Given the direction of mutational effects, this is regarded as a negative allelic interaction: the maternal allele is suppressing the paternal allele. This is quite unexpected, since the deleted region, the Peg3-DMR, is silenced by DNA methylation and thus presumably non-functional. Second, reactivation of the maternal allele resulted in slight down-regulation of the paternal allele (**Fig 3** and **Fig 4B**). This could be the result of negative feedback regulation [23], in which the additional expression of *Peg3* from the activated maternal allele represses the paternal allele *in trans*. Third, the paternal deletion resulted in either no or slight down-regulation of the maternal allele (**Fig 5B**). This might be regarded as a positive allelic interaction since deletion of the paternal allele further down-regulated the promoter activity on the maternal allele. However, this interpretation requires additional scrutiny in the future since the levels of the down-regulation were very marginal. Overall, these trans-allelic effects were readily detectable from the *Peg3* locus, especially from the maternal allele to paternal allele. This is quite intriguing, since allelic interaction is still rare among the genes in diploid organisms. The detection of these trans-allelic interactions, thus, underscores the complex nature of the regulatory mechanisms governing the *Peg3* imprinted locus.

Among the potential allelic interactions, the first one predicted through maternal deletion of the Peg3-DMR stands out the most based on the following reasons. First, the deleted region, the Peg3-DMR, is predicted to be non-functional due to DNA methylation, yet its deletion resulted in the most robust effects, 2-fold up-regulation of two paternally expressed genes, *Peg3* and *Usp29*. This seemingly inactive region may act as a strong suppressor for the paternal allele, although the actual mechanisms are currently unknown (**Fig 6**). Second, the trans-allelic effects were detected evenly throughout the entire body of 14.5-dpc embryos and also from one-day-old neonates. Similar up-regulation was also previously observed from the organs of adult mice, including fat and kidney [17]. This indicates that the observed trans-allelic effects are consistent throughout the different stages of development and among various tissues. These trans-allelic effects appeared to be also uniform in terms of the levels of up-regulation, mostly 2-fold in the tissues tested so far. This may be an indication that the observed up-regulation may be an outcome of the trans-allelic events occurring during the early stages of embryogenesis. It is reasonable to predict that some mechanisms controlling the proper gene dosage of the *Peg3* locus may require both alleles of the Peg3-DMR, in particular the maternal allele. Yet, deleting this critical region might result in incorrect epigenetic modifications on the remaining paternal allele, subsequently causing the consistent and uniform up-regulation. Overall, it should be of great interest to pursue this possibility in the near future given the unique nature of the observed trans-allelic effects.

The observed trans-allelic effects are mainly associated with some changes within the genomic region of the Peg3-DMR. The inversion, interestingly, did not cause any impact, suggesting that the orientation of this region may not be a critical factor (**Fig 4C**). According to the previous studies, deletion of the 2.5-kb YY1 binding region within the 4-kb Peg3-DMR also caused similar trans-allelic effects, exhibiting an increased growth rate among the animals with the maternal deletion of this YY1 binding region [16]. Furthermore, another mutant allele targeting 7 YY1-binding sites also displayed similar trans-allelic effects, resulting in the slight up-regulation of *Peg3* among the animals with the maternal transmission [24]. Given these observations, it is likely that the trans-allelic effects may be related to some unknown features that are associated with this 2.5-kb genomic region. This 2.5-kb genomic region is very unusual: it has an unusually large number of YY1 binding sites, 10 to 15 YY1 binding sites among individual mammals, and also maintains tandem repeat sequence structure [25, 26]. It has been perplexing why this Peg3-DMR has maintained these unusual features during mammalian evolution. These features might be designed for the observed trans-allelic interaction. For instance, the maternal allele of this 2.5-kb YY1 binding region might regulate the paternal allele in a paramutation-like mode by providing some unknown small transcripts that could be involved in epigenetic modifications [9]. Also, this 2.5-kb YY1 binding region might be the genomic region that recruits the two alleles of the *Peg3* locus to the same compartments or space within the nucleus for transcription [27], thus allowing an opportunity to communicate trans-allelically in a transvection-like mode [10, 11]. Taken together, it would be very exciting to test whether these features play some roles in this unexpected trans-allelic interaction.

## Materials and methods

### Ethics statement

All the experiments related to mice were performed in accordance with National Institutes of Health guidelines for care and use of animals, and also approved by the Louisiana State University Institutional Animal Care and Use Committee (IACUC), protocol #16–060.

### Mouse breeding

In the current study, we used the following mutant strains: *Peg3*^*CoKO/+*^, *Peg3*^*KO2/+*^, *Peg3*^*U1Δ/+*^, *Peg3*^*Inv/+*^ [17–21]. We performed six individual sets of breeding experiments with these mutant strains. The pups from each breeding experiment were analyzed in terms of sex, genotype and weight. For genotyping, genomic DNA was isolated from either clipped ears or tail snips by incubating the tissues overnight at 55°C in the lysis buffer (0.1 M Tris-Cl, pH 8.8, 5 mM EDTA, pH 8.0, 0.2% SDS, 0.2 M NaCl, 20 μg/ml Proteinase K). The isolated DNA was subsequently used for genotyping. The sex of the pups was determined through PCR using the following primer set: mSry-F (5’-GTCCCGTGGTGAGAGGCACAAG-3’) and mSry-R (5’-GCAGCTCTACTCCAGTCTTGCC-3’). The information regarding individual primer sequences for each mutant allele is available through previous studies [17–21].

### β-Gal staining

β-galactosidase activity was visualized in whole-mount embryos as previously described [28]. Briefly, embryos were harvested at 14.5-dpc from time-mated dams. The embryos were removed from the embryonic sac and washed in 1X PBS and then fixed by immersing in 4% paraformaldehyde for 1.5–2 hours. Embryonic sacs were used for PCR-based genotyping and sex determination. After fixation, embryos were washed twice in 1X PBS for 10 minutes each and then permeabilized by rinsing three times 10 minutes each in detergent solution (PBS/2mM MgCl2/0.01% Sodium deoxycholate/0.02% NP-40). Staining was performed 2–16 hours with periodic checking at 37°C in staining solution (PBS/2mM MgCl2/0.01% sodium deoxycholate/0.02% NP-40/5mM potassium ferricyanide/5mM potassium ferrocyanide) supplemented with 1 mg/ml of X-gal. Rinsing in PBS stopped the staining reaction. Whole-mount embryos were visualized in natural sunlight and images were captured with the iPhone 6s camera.

### qRT-PCR

Total RNA was isolated from the head portion of one-day-old neonates using a commercial kit (Trizol, Invitrogen). The total RNA was reverse-transcribed using the M-MuLV kit (Invitrogen), and the subsequent cDNA was used as a template for quantitative real-time PCR. This analysis was performed with the iQ SYBR green supermix (Bio-Rad) using the *ViiA 7 Real-Time PCR* System (*Life Technologies*). All qRT-PCR reactions were carried out for 40 cycles under standard PCR conditions. The analyses of the results from qRT-PCR were described previously [17–21]. Statistical significance of potential difference of expression levels of a given gene between two samples was tested with Mann-Whitney U test. The information regarding individual primer sequences is available through previous studies [29].
